# Cuproptosis regulator-mediated patterns associated with immune inﬁltration features and construction of cuproptosis-related signatures to guide immunotherapy

**DOI:** 10.3389/fimmu.2022.945516

**Published:** 2022-09-29

**Authors:** Gongjun Wang, Ruoxi Xiao, Shufen Zhao, Libin Sun, Jing Guo, Wenqian Li, Yuqi Zhang, Xiaoqian Bian, Wensheng Qiu, Shasha Wang

**Affiliations:** ^1^ Department of Oncology, Affiliated Hospital of Qingdao University, Qingdao, China; ^2^ Shandong Cancer Hospital and Institute, Shandong First Medical University and Shandong Academy of Medical Sciences, Jinan, China

**Keywords:** liver hepatocellular carcinoma, cuproptosis, risk signature, immunotherapy, prognosis

## Abstract

**Background:**

Liver hepatocellular carcinoma (HCC) is a prevalent cancer that lacks a sufficiently efficient approach to guide immunotherapy. Additionally, cuproptosis is a recently identified regulated cell death program that is triggered by copper ionophores. However, its possible significance in tumor immune cell infiltration is still unclear.

**Methods:**

Cuproptosis subtypes in HCC were identified using unsupervised consensus cluster analysis based on 10 cuproptosis regulators expressions, and a cuproptosis-related risk signature was generated using univariate and LASSO Cox regression and validated using the ICGC data. Moreover, the relationship between signature and tumor immune microenvironment (TME) was studied through tumor immunotherapy responsiveness, immune cell infiltration, and tumor stem cell analysis. Finally, clinical specimens were analyzed using immunohistochemistry to verify the expression of the three genes in the signature.

**Results:**

Two subtypes of cuproptosis regulation were observed in HCC, with different immune cell infiltration features. Genes expressed differentially between the two cuproptosis clusters in the TCGA were determined and used to construct a risk signature that was validated using the ICGC cohort. Greater immune and stromal cell infiltration were observed in the high-risk group and were associated with unfavorable prognosis. Elevated risk scores were linked with higher RNA stemness scores (RNAss) and tumor mutational burden (TMB), together with a greater likelihood of benefitting from immunotherapy.

**Conclusion:**

It was found that cuproptosis regulatory patterns may play important roles in the heterogeneity of immune cell infiltration. The risk signature associated with cuproptosis can assess each patient’s risk score, leading to more individualized and effective immunotherapy.

## Introduction

Liver cancer is an aggressive tumor with poor outcomes (1). Liver hepatocellular carcinoma (HCC) accounts for more than 80% of all primary liver malignancies for which surgical resection is currently the most effective treatment (2). Unfortunately, diagnosis of HCC is frequently delayed leading to unfavorable outcomes (3). Despite recent significant progress in immunotherapy and targeted therapy, the five-year survival rate remains low, at approximately 15% (4). An important reason for this is that patients with HCC vary in their response to immunotherapy, and the factors that influence and predict the response to immunotherapy in HCC remain unclear (5).

Recent research has shown that copper toxicity-mediated cell death differs from other forms of regulated cell death; this novel mechanism is termed cuproptosis (6). Copper is both necessary and potentially toxic for cells. It is an essential cofactor required by all living organisms to function properly (7–9); however, high levels of copper accumulation or improper distribution in the cell can lead to cell death. Imbalances in copper homeostasis in cells can lead to severe disease in humans, including tumor development (10, 11). Excess copper has been linked with various types of cancer, including breast (12–14), prostate (15–17), colon (18), lung (19), brain (20), and liver (21) cancer. However, the reasons underlying elevated copper levels in tumors are unclear. Recent studies revealed the mechanism associated with copper-mediated cell death. A study by Tsvetkov et al. showed that cuproptosis is associated with copper binding to fatty acylated moieties of tricarboxylic acid, resulting in the abnormal aggregation of fatty acylated proteins and the loss of iron-sulfur cluster proteins, leading to proteotoxic stress and cell death (6). These findings suggest a starting point for investigating the application of cuproptosis in disease treatment, especially, for tumor therapy.

Copper, as a key trace element, is necessary for the functioning of the immune system. Copper deficiency adversely affects immune function and exposes the organism to microbial infection (22). The immune system requires copper for a variety of functions. The metal can modulate the activation of cells associated with innate immunity such as macrophages and neutrophils during bacterial infection and leukocyte differentiation, maturation, and migration (23, 24). Copper deficiency may also affect immune cell distribution in tissues or the maturation pattern of leukocyte populations (25). In addition, intratumoral copper can regulate PD-L1 expression and affect tumor immune escape (26). These reports suggest an association between copper and the modulation of the immune response.

Here, we used data from the TCGA to investigate cuproptosis subtypes and correlate these subtypes with immune-infiltration features. Signature genes associated with cuproptosis were identified by differential and prognostic analysis, and a risk signature using these genes was generated. This cuproptosis-related risk signature identifies specific features of immune infiltration, permitting the development of individualized immunotherapy.

## Methods

### Collection of data

Data on gene expression (fragments per kilobase, FPKM), mutations, and clinical data were obtained from the Cancer Genome Atlas (TCGA) database (https://portal.gdc.cancer.gov/). Patients lacking survival information were not enrolled. Xena (http://xena.ucsc.edu/) was utilized to obtain copy number variation (CNV) data and RNA stemness scores (RNAss) for TCGA samples. Validation was performed using data available from the International Cancer Genome Consortium (ICGC) (https://dcc.ICGC.org/projects/LIRI-JP) for 229 HCC patients with complete information. The 10 cuproptosis regulators identified by Tsvetkov et al. (6) were used (**Table S1**). Since TCGA and ICGC data are publicly available, permission from an ethics committee was not necessary for this work. Despite this, the investigation was performed in conformity with the procedure specifications established by the TCGA and the ICGC.

### Profiling of cuproptosis regulators

Principal component analysis (PCA) using the expression of cuproptosis regulators was performed on both HCC and normal samples with the package “scatterplot3d” in R, with relationships between the regulators examined using “corrplot”, and the CNVs of 10 regulators on human chromosomes using “Rcircos”.

### Consensus cluster analysis of cuproptosis regulators

The “ConsensusClusterPlus” package in R was employed for unsupervised consensus cluster analysis (27). The criteria for clustering were an initial gradual increase in the cumulative distribution function (CDF) curve, strong intraclass relationships and weak interclass relationships, and no unacceptably small sample sizes in any of the groups. The R packages “ggplot2” and “Rtsne” were used for PCA and t-distributed random neighbor embedding (t-SNE) analysis, respectively. Overall survival (OS) in the different clusters was analyzed with Kaplan-Meier (K-M) curves. Enrichment analysis of genes was conducted with gene set enrichment analysis (GSEA, v4.2.1; https://www.gsea-msigdb.org/gsea/downloads.jsp) (28) and Gene Ontology (GO) using the R package “clusterProfiler” (29).

### Immune infiltration profiles in the different groups

The relative abundance levels of immune cell types in the HCC microenvironment were examined by single-sample GSEA (ssGSEA) (30, 31). Immune cell genomes obtained from Charoentong et al. (32) were used as markers for the various cell types. Tumor immune dysfunction and rejection (TIDE; http://tide.dfci.harvard.edu/) was also used as a marker for examining immune escape mechanisms and predicting the response to immunotherapy (33). Higher TIDE scores indicate a greater likelihood of immune escape, leading to the probability of reduced treatment efficacy. The degree of infiltration of six immune cell types associated with the expression of three differentially expressed genes (DEGs) was determined with the Tumor Immune Estimation Resource (TIMER) database (https://cistrome.shinyapps.io/timer/) (34, 35). The “SCNA” module was utilized to compare infiltration levels between tumors with different CNVs for specific genes.

### Comparison of responsiveness to immunotherapy

The IMvigor 210 cohort comprising cancer patients treated with programmed death ligand 1 (PD-L1) inhibitors was used for the prediction of immunotherapy response (36). These samples were categorized based on the patient’s response, namely partial response (PR), complete response (CR), progressive disease (PD), and stable disease (SD), with PR and CR representing immunotherapy response and PD and SD representing a lack of response. The R package “pRRophetic” was utilized to compute the half-maximal inhibitory concentration (IC50) of the drugs with low IC50 values indicating greater drug sensitivity.

### Construction and validation of the HCC risk signature

Two patient clusters were formed based on the expression of the 10 cuproptosis-related regulatory genes. Genes expressed differentially between the clusters were determined with the empirical Bayesian method in the “limma” package with significance set as an adjusted P-value < 0.0001. Further DEGs between HCC samples and adjacent normal tissue were analyzed to narrow down candidate signature genes (P<0.0001). Finally, univariate Cox regression as well as least absolute shrinkage and selection operator (LASSO) analysis (37) were employed to detect DEGs in the final signature. Risk scores were calculated for all HCC samples and groups with low-risk and high-risk were identified in the TCGA cohort based on median risk scores. The risk score was calculated as:


$$\text{Risk Score }=\sum_{i=0}^{n}\beta i*Gi$$

where *βi* denotes the gene coefficient; *i* and *n* are the number of genes in the signature; *Gi* denotes the gene expression value.

The risk scores for samples in the ICGC set were then determined using the risk coefficients of the signature DEGs in the TCGA, and the ICGC samples were allocated two groups (low risk and high risk) using the risk thresholds observed in the TCGA. K-M survival curves were utilized to evaluate OS between the groups. Risk signature sensitivity and specificity were calculated using receiver operating characteristic (ROC) curves.

### Nomogram construction and verification

In both the TCGA and ICGC cohorts, hazard ratio models were developed using univariate and multivariate Cox regression to detect independent prognostic variables. A nomogram including risk scores and other clinicopathological features was then constructed in the TCGA cohort and one-, three-, and five-year calibration curves for constructed for accuracy validation. The area under the ROC curve (AUC) and decision curve analysis (DCA) were used to evaluate the nomogram’s discriminative power.

### Determination of the relationships between tumor mutation and risk signature

The R package “maftools” was used to generate the waterfall charts for the low- and high-risk groups respectively. Somatic mutation analysis was used to determine the tumor mutational burden (TMB) score of each TCGA-HCC patient. Subsequently, the Pearson correlation analysis was studied to determine the link between risk score and TMB. The data was further expressed in scatterplots and boxplots. In addition, boxplots were generated to demonstrate the differences in immune cell infiltration between the low- and high-TMB groups, respectively. The overall survival (OS) rate among these groups were determined through K-M survival curves.

### Comparison of immunohistochemistry in normal and HCC samples

Immunohistochemical staining was performed with antibodies against human G6PD (25413-1-AP; proteintech), CDCA8 (bs-7834R; Boaosen), and Cyclin B1 (bs-0572R; Boaosen), followed by a pathologist based on the percentage of positive cells and staining intensity to score sections. Staining intensity was scored as 0 (negative), 1 (weak), 2 (moderate), or 3 (strong), and the proportion of positive cells expressed was scored as 1 (0-25%), 2 (26-50%), 3 (51– 75%) or 4 (76–100%). The final score was obtained by multiplying the expression ratio and the signal intensity.

### Expression and prognostic analysis of three DEGs

The expression of three genes differentially expressed between normal tissues and HCC was analyzed using the “limma” R package, and the “surv cutpoint” function in “survminer” was employed to examine the optimal cut-off expression values for survival. Groups with high and low expression were established and OS was compared between groups using K-M analysis.

### Statistical analysis

All statistical analysis was conducted using R version 4.1.2 (http://www.R-project.org) and its accompanying packages. The Kruskal-Wallis test and one-way ANOVA were used to examine differences between three or more groups. OS comparisons were conducted using K-M analysis and the log-rank test. Univariate and multivariate Cox regression analyses were used to calculate hazard ratios and identify independent risk factors. The diagnostic value of risk scores and nomogram models was assessed using ROC curves. P<0.05 was considered to be the criterion for significant statistical difference.

## Results

### The landscape of cuproptosis regulators in HCC

**Figure 1:**
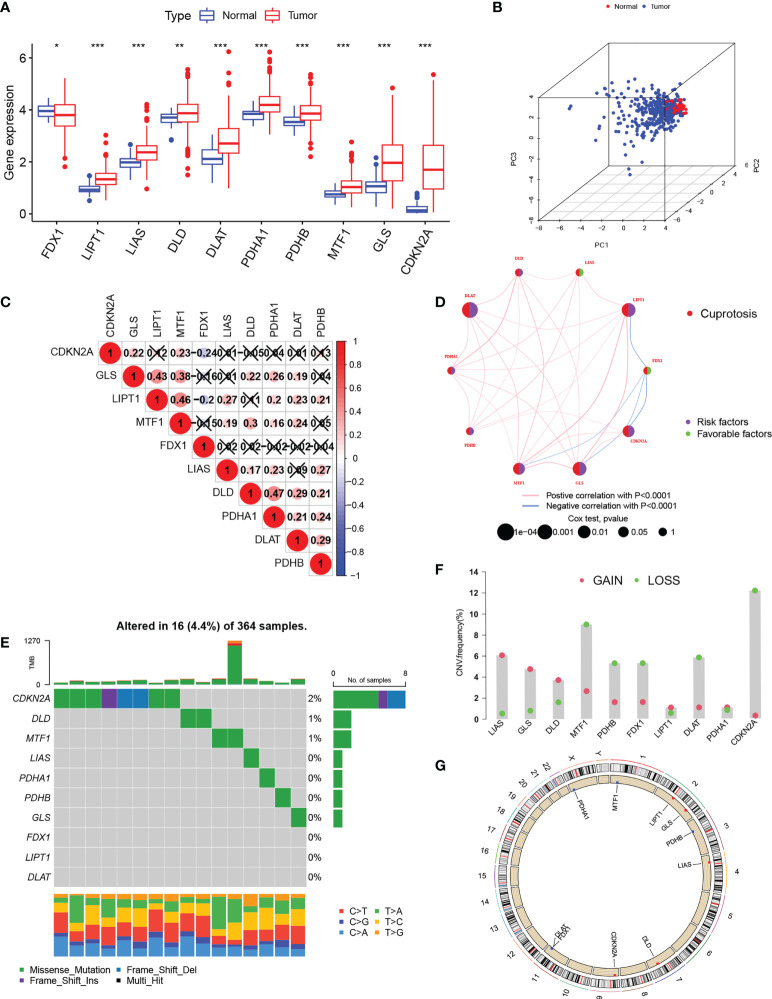
Expression of cuproptosis-regulatory genes in HCC. **(A)** Comparison of expression between normal and tumor tissues **(B)** PCA of the expression of the 10 genes. **(C)** Spearman correlations between genes. The color blue represents negative regulation; the color red represents positive regulation. **(D)** Comprehensive network map combining cuproptosis regulator interactions and prognosis. **(E)** Mutation frequencies of the 10 regulatory genes in 364 HCC s specimens. **(F)** CNV values for cuproptosis regulators in HCC specimens. **(G)** Chromosomal locations of CNV alterations in cuproptosis regulators. *P < 0.05; **P < 0.01; ***P < 0.001. HCC, hepatocellular carcinoma; PCA, principal component analysis; CNV, copy number variation.

Based on the study by Tsvetkov et al. (6),10 cuproptosis regulators (7 positive regulators and 3 negative regulators) were included in this study. The expression of these regulators differed significantly between normal and HCC tissues. Apart from the critical regulator FDX1, which was down-regulated in HCC, all other regulators were up-regulated in tumor tissue in comparison with controls (**Figure 1A**). PCA analysis indicated that the expression of all 10 regulatory genes clearly distinguished between HCC and normal tissue (**Figure 1B**). After the division of the genes into high and low expression groups using “survminer”, it was observed that low levels of FDX1 were linked with poor HCC prognosis, while the remaining genes exhibited an opposite trend (P>0.05 for DLD due to insufficient sample) (**Figure S1**). Analysis of co-expression of the regulators indicated that all had positive regulatory relationships apart from FDX1 where a negative relationship was observed (**Figure 1C**). The highest correlation (0.47) was seen between DLD and PHAR1. The regulatory network demonstrated both the interactions between the genes and their potential prognostic significance for HCC (**Figure 1D**). The inconsistent findings for FDX1 suggest that it may be more critical in the regulation of cuproptosis (6). Further analysis of the genetic basis of cuproptosis using the TMBs and CNVs of the 10 regulators showed alterations in 5.85% of the 364 samples (16 mutations). The highest number of mutations was seen in CDKN2 followed by DLD and MTF1 (**Figure 1E**). CNVs were common in the 10 genes, with LOSS occurring more frequently than GAIN. LIAS and GLS showed higher CNV gains, while CDKN2 exhibited higher loss (**Figure 1F**). **Figure 1G** shows the chromosomal location of the regulators’ CNVs. These findings marked different levels of these regulatory factors in tumor and normal tissues, implicating disturbances in cuproptosis regulator expression in HCC.

### Correlations between cuproptosis clusters and TME features

**Figure 2:**
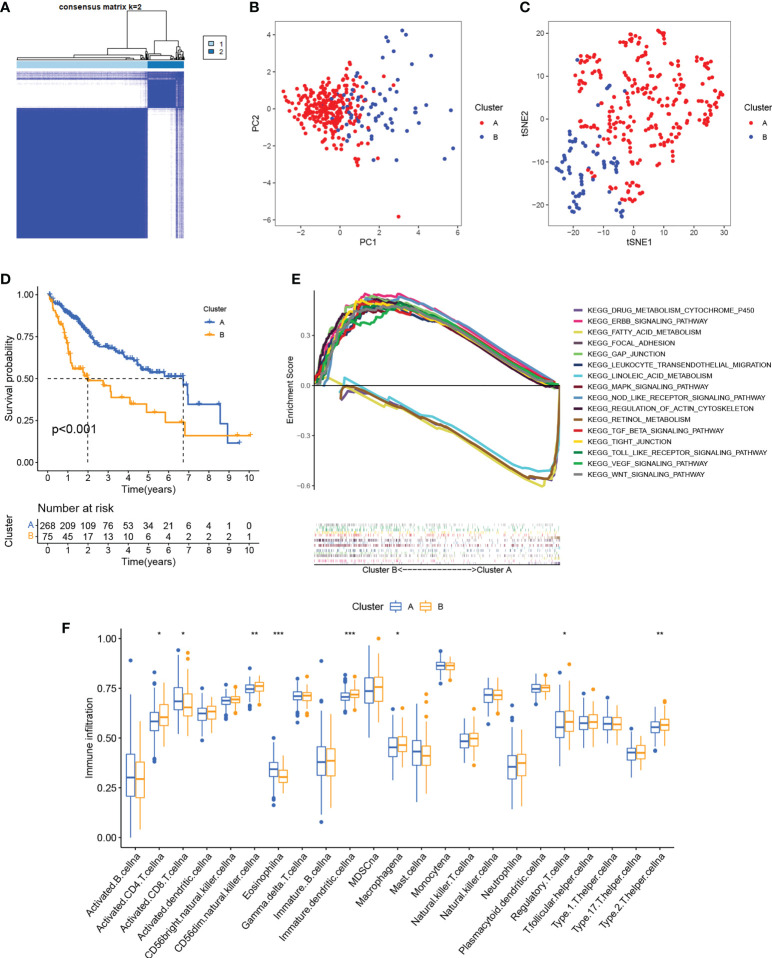
Identification of cuproptosis clusters. **(A)** Consensus matrix based on cuproptosis regulator expression in the HCC cohort at k = 2. **(B)** PCA of consensus matrix when k = 2. **(C)** T-SNE analysis of consensus matrix when k = 2. **(D)** K-M survival analysis between the two cuproptosis clusters. **(E)** Multiple GSEA analysis between the two cuproptosis clusters. **(F)** Infiltration of immune cells between the two cuproptosis clusters. *P < 0.05; **P < 0.01; ***P < 0.001. HCC, hepatocellular carcinoma; PCA, principal component analysis; T-SNE, t-distributed random neighbor embedding; K-M, Kaplan-Meier; GSEA, gene set enrichment analysis.

Patterns of cuproptosis were determined by unsupervised consensus cluster analysis of cuproptosis regulator expression in HCC and the results of the consensus clustering (k2–9) were visualized with a CDF plot (**Figures 2A**; **S2**). Examination of the consensus matrix showed that k=2 was the best option, and that each sample in the cluster exhibited a strong correlation (**Figure 2A**). The HCC patients were, therefore, assigned to one of two clusters (A or B). The clustering results are shown in **Table S2**. PCA (**Figure 2B**) and t-SNE (**Figure 2C**) analysis showed that there were two discrete directions within the clusters. Furthermore, we found that the prognosis of these patients was significantly different, with patients in cluster A showing better OS (**Figure 2D**). Multi-GSEA enrichment analysis indicated significant differences in biological processes between the clusters, specifically, in metabolism, tumor signaling pathways, and immune- and matrix-related pathways (**Figure 2E**). The enriched metabolic processes included those associated with fatty acid, linoleic acid, retinol, and drug metabolism cytochrome P450 showing greater enrichment in cluster A. Cluster B showed greater enrichment in the tumor-associated ERBB, MAPK, TGFβ, VEGF, and Wnt signaling pathways. Pathways related to immune activation were associated mostly with cluster B; these included leukocyte transendothelial migration, Toll-like receptor signaling, and NOD-like receptor signaling. We then investigated infiltrating immune cells in the clusters (**Figure 2F**), finding that the majority of immune cells were strongly associated with cluster B. At the same time, we also found that matrix-activated pathways such as focal adhesions, gap junctions, and tight junctions were enriched in cluster B (**Figure 2E**). It has been reported that tumor stromal cells are involved in immune regulation (38). Stromal cells are able to block immune cell entry into the tumor parenchyma and can also block T-cell killing of tumor cells (39). Tumor cells and stromal cells can induce angiogenesis, thereby promoting tumor metastasis (40).. In addition, regulatory T cells and CD 56bright NK cells highly expressed in cluster B can act as immunosuppressive cells to promote tumorigenic immune escape (41, 42). The results of the immune microenvironment analysis described above validate the poor prognosis of cluster B patients. Taken together, we found that the expression of cuproptosis regulatory proteins in HCC defines two patient clusters with significantly different immune infiltration features. Cluster A is an immune-desert phenotype with reduced immune activity and immune cell numbers while cluster B is an immune-exclusion phenotype with infiltration restricted to the peripheral matrix of tumor cells.

### Characterization of cuproptosis-related phenotypes

**Figure 3:**
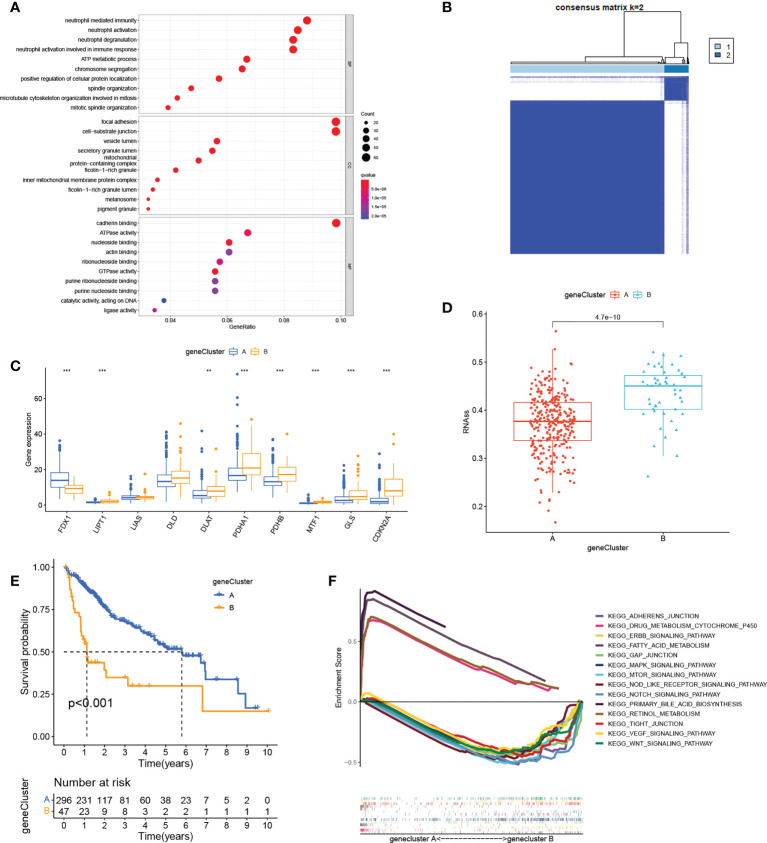
Gene cluster determination. **(A)** GO enrichment analysis of cuproptosis-related DEGs. **(B)** Consensus matrix based on cuproptosis-related gene expression in the HCC cohort at k = 2. **(C)** Differential expression of cuproptosis regulators in the two clusters. **(D)** RNAss values of cuproptosis regulators in the two clusters. **(E)** K-M survival analysis of cuproptosis regulators in the two clusters. **(F)** Multiple GSEA analysis between the two clusters. **P < 0.01; ***P < 0.001. GO, Gene Ontology; DEGs, differentially expressed genes; HCC, liver hepatocellular carcinoma; RNAss, RNA stemness scores; K-M, Kaplan-Meier; GSEA, gene set enrichment analysis.

We next identified 629 cuproptosis-associated DEGs using the R package “limma”. GO analysis showed enrichment of the DEGs in immune- and stromal-associated pathways (including neutrophil mediated immunity, neutrophil activation involved in immune response, focal adhesion, and cell−substrate junctions), confirming that cuproptosis is closely associated with the regulation of the immune microenvironment of the tumor (**Figure 3A**). We then further narrowed the range by comparing gene expression between the normal and HCC groups to obtain 30 DEGs. Univariate Cox regression yielded 27 prognosis-associated DEGs with HCC patients again divided into two clusters according to the unsupervised consensus cluster analysis of the expression of these genes (**Figures 3B**; **S3**). The gene clustering results are presented in **Table S3**. Differential expression of most of the cuproptosis regulators was visible between the clusters (**Figure 3C**). Additionally, RNAss values were significantly increased in gene cluster B compared with gene cluster A (**Figure 3D**), demonstrating the adverse effects of high RNAss values on HCC prognosis (**Figure 3E**). Oncogenic and immune- and matrix-activated pathways were enriched in cluster B, while cluster A showed enrichment in metabolic pathways (**Figure 3F**). These findings suggest the presence of two cuproptosis-associated regulatory patterns in HCC.

### Development of the cuproptosis-associated risk signature

**Figure 4:**
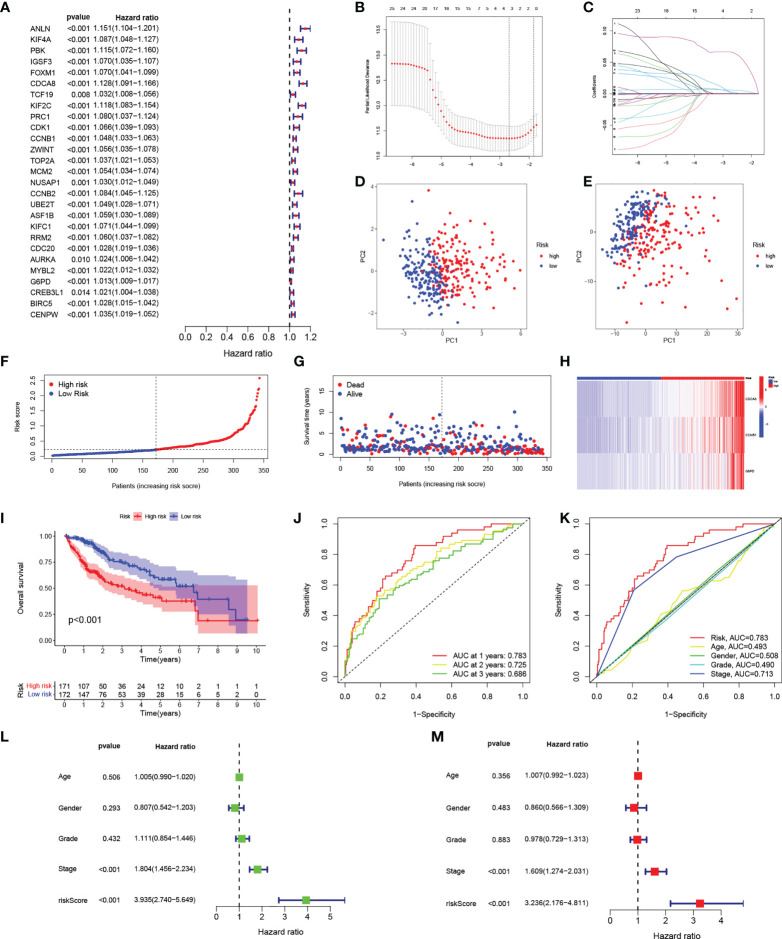
Construction of the risk signature. **(A)** Forest plot of univariate Cox regression results for the 27 DEGs. **(B)** Cross-validation for selection of tuning parameters in LASSO regression. **(C)** LASSO coefficient profiles of candidate DEGs. **(D)** PCA of samples from high- and low-risk groups. **(E)** PCA of DEGs in the high- and low-risk groups. **(F)** Risk score distribution of HCC patients in the TCGA cohort. **(G)** Scatter plot of survival status of HCC patients in the TCGA cohort. **(H)** Heatmap of the expression of the three signature genes in high- and low-risk populations in the TCGA cohort. **(I)** K-M curves of OS in HCC patients in the risk score-based TCGA cohort. **(J)** ROC curves of prognostic signatures for one, two, and three years in the TCGA cohort. **(K)** ROC curves of prognostic signatures and other clinicopathological features for one year in the TCGA cohort. **(L, M)** Forest plots of univariate and multivariate Cox regression analyses of prognostic signatures and clinical features in the TCGA cohort. DEGs, differentially expressed genes; LASSO, least absolute shrinkage and selection operator; PCA, principal component analysis; HCC, hepatocellular carcinoma; TCGA, the Cancer Genome Atlas; K-M, Kaplan-Meier; OS, overall survival; ROC, receiver operating characteristic.

The above analysis was performed on a patient population. We then investigated the precise quantification of cuproptosis patterns in individual patients. Using the 27 genes identified in the univariate analysis (**Figure 4A**), we performed LASSO to prevent signature overfitting (**Figures 4B, C**). The risk signature was finally constructed using three signature genes (CDCA8, CCNB1, and G6PD). The risk score assigned to each sample was determined as: Risk Score = (0.066092028117448) × the CDCA8 expression + (0.00250484775117196) × the CCNB1 expression + (0.00581254249797367) × the G6PD expression. This enabled the HCC patients to be entirely separated into groups with low and high risk according to the median risk score. The risk score results are provided in **Table S4**. PCA showed the clear separation of the 343 HCC patients and 279 DEGs in two independent clusters, trending in two different directions (**Figures 4D, E**). The scatter plots of risk scores and patient survival statistics are presented in **Figures 4F, G**. As depicted in the figure, a higher risk score was related to both decreased survival and greater mortality. The heatmap (**Figure 4H**) shows the expression of the three DEGs in the signature in the TCGA cohort (**Figure 4H**). K-M curves indicated higher OS rates in the low-risk group (**Figure 4I**). To examine the performance of signature, ROC curves for one-, two-, and three-year OS were generated, with AUCs of 0.783, 0.725, and 0.686, respectively (**Figure 4J**). The risk signature’s superiority was further established by comparing the one-year ROC curves with other clinicopathological features (**Figure 4K**). Univariate Cox regression was employed to examine associations, finding that stage (HR = 1.804, 95% CI = 1.456–2.234, p < 0.001) and risk score (HR = 3.935, 95% CI =2.740–5.649, p < 0.001) were related to OS in the TCGA set (**Figure 4L**). Multivariate regression verified that stage (HR = 1.609, 95% CI = 1.274–2.031, p < 0.001) and risk score (HR = 3.236, 95% CI = 2.176–4.811, p < 0.001) were independent prognostic factors for OS (**Figure 4M**).

### Validation of the risk signature

**Figure 5:**
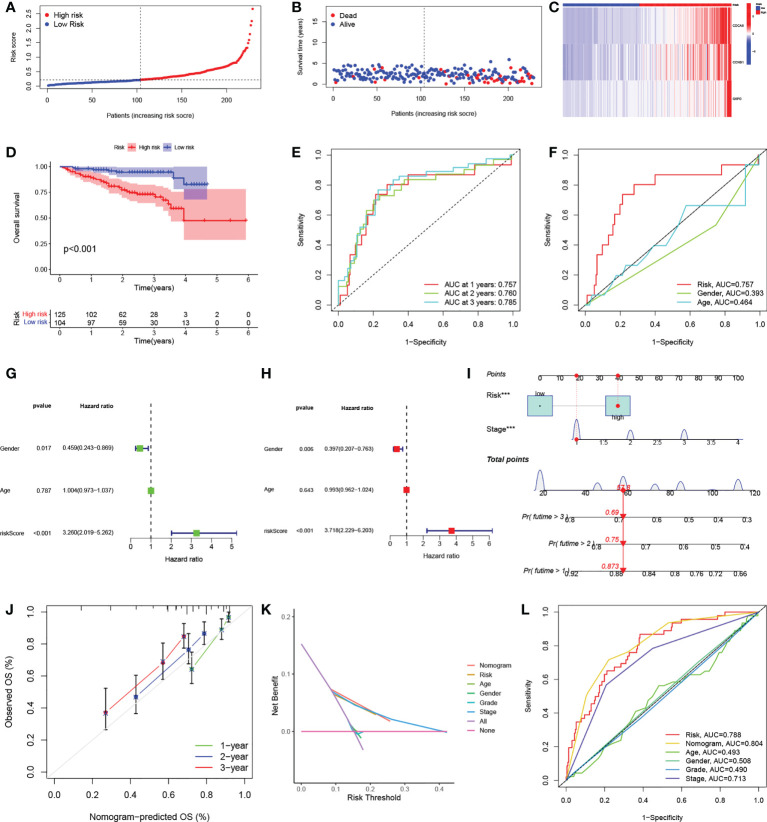
Independent verification of risk signatures and construction of nomograms. **(A)** Distribution of HCC patient risk scores in the ICGC cohort. **(B)** Scatterplot showing HCC patient OS in the ICGC cohort. **(C)** Heatmap of the expression of the three signature genes in high- and low-risk groups in the ICGC cohort. **(D)** K-M curves for HCC patient OS in the risk score-based ICGC cohort. **(E)** ROC curves of one-, two, and three-year prognostic signatures in the ICGC cohort. **(F)** ROC curves of one-year prognostic signatures with other clinicopathological characteristics in the ICGC cohort. **(G-H)** Forest plots of univariate and multivariate Cox regression analyses of prognostic signatures and other clinicopathological features in the ICGC cohort. **(I)** Nomogram combining risk signatures and clinical stages for OS prediction in the TCGA cohort. **(J)** Calibration plots for nomograms at one, three, and five years. **(K)** DCA of the nomogram, risk signature, and other clinicopathological features. **(L)** ROC curves of the nomogram, risk signature, and clinicopathological characteristics at one year. HCC, hepatocellular carcinoma; ICGC, International Cancer Genome Consortium; K-M, Kaplan-Meier; OS, overall survival; ROC, receiver operating characteristic; DCA, decision curve analysis.

The test group samples from the ICGC were divided into low- (n = 104) and high- (n = 125) risk score groups using the cutoff values determined in the TCGA cohort. **Figures 5A, B** show the risk curves and scatterplots while the heatmap in **Figure 5C** illustrates the expression patterns of the three signature DEGs in the ICGC set. The high-risk group, as determined in the TCGA cohort, had a worse overall prognosis (**Figure 5D**). The AUCs of the ROC curves for one, two, and three years were 0.757, 0.760, and 0.785, respectively (**Figure 5E**), and the AUC of the risk score at one year was larger than those for other clinical parameters (**Figure 5F**). Both univariate and multivariate regression analyses found that the P-values for both risk score and sex were below 0.05, demonstrating that signature functioned as an independent predictor of HCC prognosis in the ICGC set, and further confirming its reliability in the TCGA set (**Figures 5G, H**).

### Nomogram creation and validation

To further improve the clinical utility of risk signature, the identified independent prognostic risk factors, namely, risk score and stage, were utilized to develop a nomogram for predicting OS in the TCGA cohort (**Figure 5I**). The calibration curves at one, three, and five years showed that the nomogram accurately predicted the outcomes of HCC patients (**Figure 5J**). In addition, the DCA curve showed that the nomogram had specific advantages over other clinical parameters (**Figure 5K**). The AUCs for the ROC curves suggested that the nomogram, with an AUC of 0.804, was superior to age (AUC=0.493), sex (AUC=0.508), pathological grade (AUC=0.490), TNM stage (AUC=0.713), and risk signature (AUC=0.788) for predicting prognosis in HCC patients (**Figure 5L**).

### Relationships between the risk signature and immune characteristics, clinical parameters

**Figure 6:**
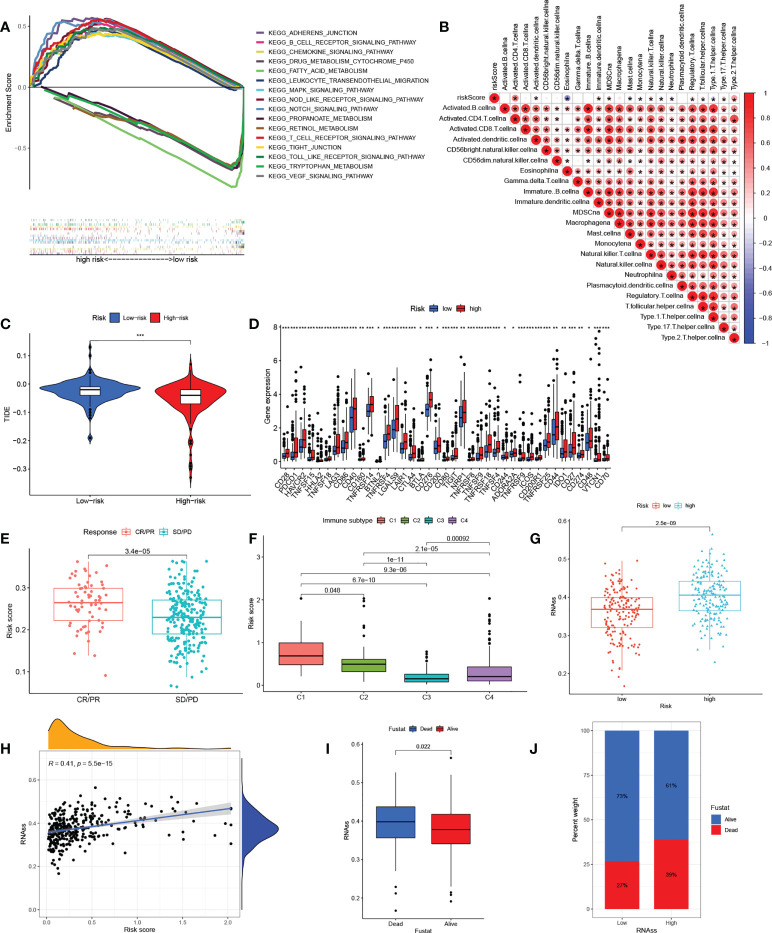
The correlation between the immunity and risk signature. **(A)** Multiplex GSEA analysis between groups with high and low risk **(B)** Correlations between infiltration of immune cell levels and risk scores. The color blue implies a negative association; the color red denotes a positive association. **(C)** TIDE scores in the groups with high and low risk. **(D)** Expression of immune checkpoints in the groups with high and low risk. **(E)** Risk scores in anti-PD1/PD-L1-treated CR/PR samples and SD/PD samples in the IMvigor210 cohort. **(F)** Risk scores in relation to immune cell subtypes. **(G)** RNAss values in the groups with high and low risk. **(H)** Relationships between risk scores and RNAss values. **(I)** RNAss values in deceased and surviving patients. **(J)** Proportions of deceased and surviving patients in the groups with high and low RNAss. GSEA, gene set enrichment analysis; TIDE, tumor immune dysfunction and rejection; PR, partial response; CR, complete response; PD, progressive disease; SD, stable disease; RNAss, RNA stemness scores. The asterisks represented the statistical p value (*P < 0.05; **P < 0.01; ***P < 0.001.)

A GSEA analysis was next used to investigate physiology-related differences between the groups (**Figure 6A**). The results were consistent with those of the clustering analysis, showing that many metabolism-related pathways, including those associated with fatty acid, cytochrome P450, and retinol metabolism were enriched in the low-risk group. Tumor-associated pathways (including the MAPK, NOTCH, and VEGF pathways) and immune- and matrix-related pathways (including the Toll-like receptor, B cell receptor, T cell receptor, chemotaxis factor pathways, and attachment junctions) were associated with high risk. To confirm the immune characteristics of the signature, we examined associations between immune cells and risk scores. This indicated a positive relationship between risk scores and most immune cells (**Figure 6B**). Taken together, it appears that high-risk scores are indeed associated with increased stromal activity and immune infiltration. Interestingly, the TIDE analysis indicated that immune escape was likely to be reduced in the high-risk group after immunotherapy (**Figure 6C**). It was also found that immune checkpoint levels, including those of PDCD1 and CTLA4, were raised in the high-risk group, which thus had a greater chance of benefitting from immunotherapy (**Figure 6D**). Calculation of the risk scores of patients in the IMvigor210 set using the TCGA set risk signature indicated that patients responding (CR/PR) to anti-PD1/PD-L1 therapy showed significantly raised risk scores (**Figure 6E**). We then compared HCC samples for differences in risk scores based on tumor immunophenotyping established by Thorsson et al. (43). The immunophenotyping of the HCC samples is presented in **Table S5**. The Wound Healing (immune C1) risk score was found to be higher than others, suggesting that high-risk scores were associated with matrix activation (**Figure 6F**). In addition, analysis of tumor stemness indicated that RNAss was raised in the high-risk group (**Figure 6G**) with a positive association between the risk scores and RNAss (**Figure 6H**). The RNAss values of the HCC samples are presented in **Table S6**. Deceased HCC patients had higher RNAss values than surviving patients (**Figure 6I**). The OS values in the high- and low-RNAss groups were 61% and 73%, respectively (**Figure 6J**), indicating both the prognostic significance of RNAss and the reliability of the risk score measure. Furthermore, the associations between clinical features and risk scores are shown in **Figures S4A–F**. Risk scores were found to increase significantly with TNM stage from I to III, T stage from I to III, grade from G1 to G4, and AFP expression from low to high. The risk scores did not change significantly in response to age or sex. In addition, boxplots were used to show the results for 12 drug sensitivities by estimating IC50 values between the groups. Patient groups at low risk were significantly more sensitive to Gefitinib, Sorafenib, Nilotinib, Dasatinib, Erlotinib, and Metformin (**Figure S5A–F**), in contrast to high-risk patients who responded to Bleomycin, Doxorubicin, Gemcitabine, Tipifarnib, Imatinib, and Mitomycin.C (**Figure S5G–K**). This has reference significance for guiding the clinical medication of HCC treatment.

### Crosstalk among cuproptosis clusters, gene clusters, risk signature, and clinicopathological features

**Figure 7:**
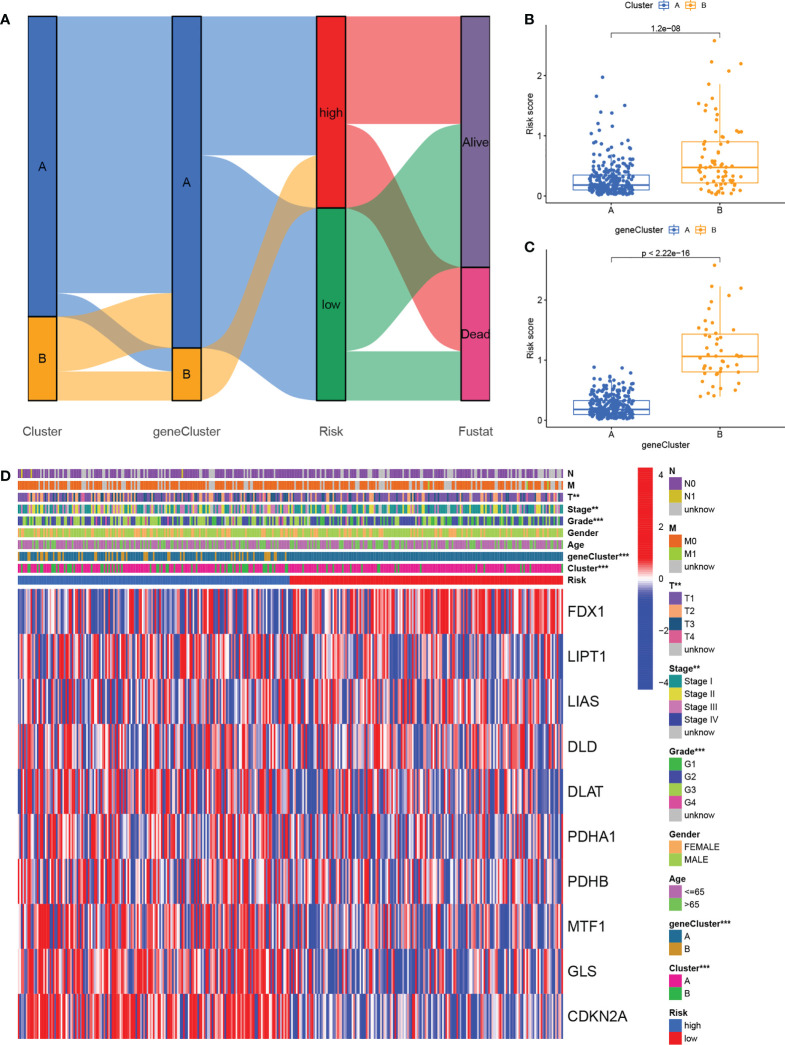
Relationship cross-links of cuproptosis clusters, gene clusters, risk signatures, and clinical features. **(A)** Alluvial diagram showing cuproptosis clusters, gene clusters, risk grouping, and survival status. **(B)** Risk scores in the cuproptosis clusters. **(C)** Risk scores in the gene clusters. **(D)** Heatmaps showing the integration of cuproptosis clusters, gene clusters, clinicopathological characteristics, and cuproptosis regulators’ expression in relation to risk groups.

The alluvial map shown in **Figure 7A** illustrated the crosstalk among cuproptosis clusters, gene clusters, and risk scores. Higher risk scores were associated with gene cluster B rather than gene cluster A (**Figure 7B**) and the cuproptosis cluster B also had higher risk scores than cluster A (**Figure 7C**). The prognosis was significantly enhanced in both cluster A and gene cluster A in comparison with their respective B clusters, again confirming the reliability and consistency of the analysis. **Figure 7D** shows a comprehensive heatmap of risk scores in relation to clinical features (including age, sex, grade, and TNM, T, N, and M stages), cuproptosis clustering, gene clustering, and cuproptosis regulator expression. A strong association between cuproptosis clusters, gene clusters, and risk signatures can be observed.

### An examination of the correlation between the risk signature and genetic mutations

**Figure 8:**
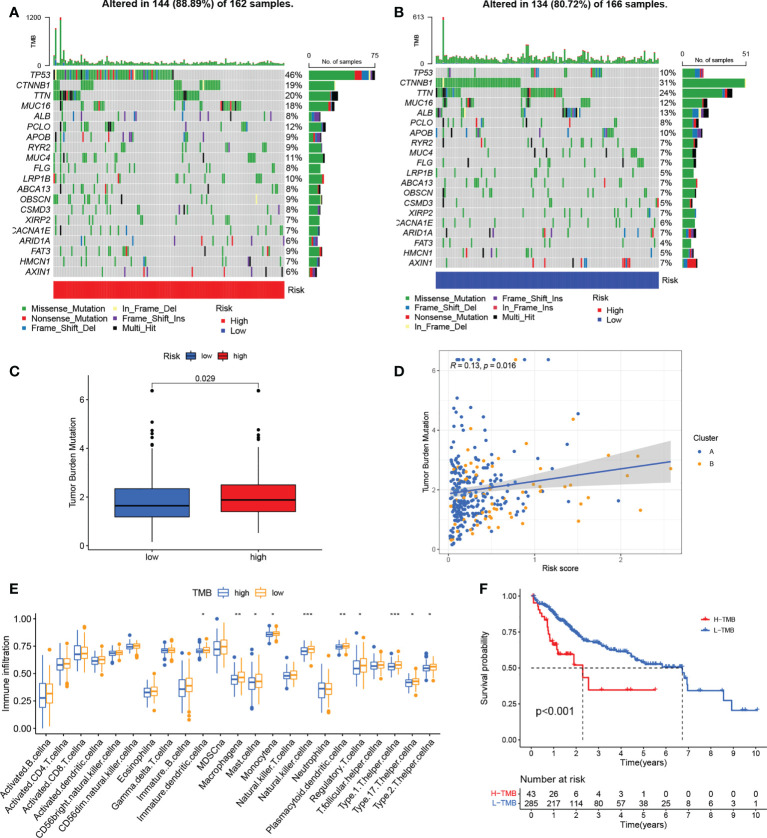
Associations between risk signatures and gene mutations. **(A, B)** Mutation frequencies in the groups with high and low risk **(C)** TMB values in the groups with high and low risk **(D)** Association between risk scores and TMB. **(E)** Infiltration of immune cell in the groups with high and low TMB. **(G)** K-M curves for OS in the groups with high and low TMB. *P < 0.05; **P < 0.01; ***P < 0.001. TMB, tumor mutational burden; K-M, Kaplan-Meier.

Differences in somatic mutation distribution between low- and high-risk scores in the TCGA set were investigated with the “maftools” package. As **Figures 8A, B** show, the TMB was greater in the group with the high-risk scores, together with an 88.89% mutation rate (altered in 144 of 162 samples) versus 80.72% for the low-scoring group (altered in 134 of 166 samples). Quantitative analysis confirmed that tumors with high scores were correlated with higher TMB values (**Figure 8C**) and that risk scores and TMB values were significantly positively correlated (**Figure 8D**). It has been reported that high TMB is related to long-term clinical sensitivity to anti-PD1/PD-L1 therapy (44), confirmed by the findings shown in **Figures 6C–E**. This suggests that curoptosis-associated variations in tumors may be critical for the anti-PD-1/PD-L1 therapeutic response. High TMB values were also linked to reduced immune cell infiltration in HCC (**Figure 8E**) while K-M survival curves showed an association between elevated TMB and worse OS (**Figure 8F**), indirectly confirming the effectiveness of the risk score in the prediction of immunotherapy outcome.

### Three DEGs in the risk signature

**Figure 9:**
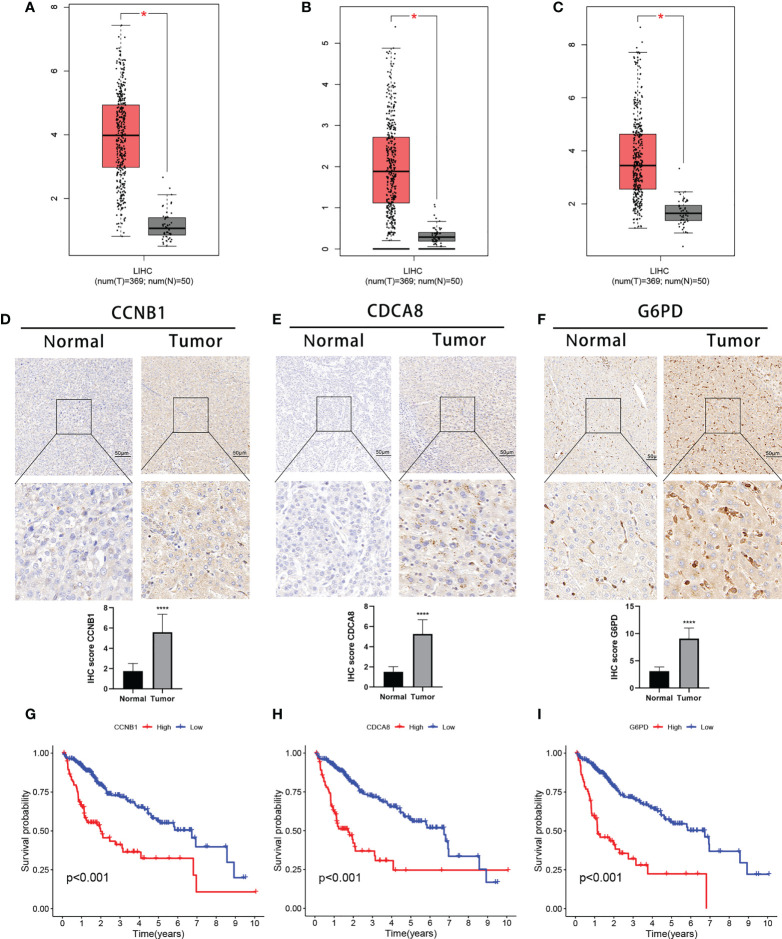
Expression of the three signature DEGs and their impact on prognosis. Expression of CCNB1 **(A)**, CDCA8 **(B)**, and G6PD **(C)** in tumor and normal samples (GEPIA). IHC analysis of CCNB1 **(D)**, CDCA8 **(E)**, and G6PD **(F)** expression in tumor and normal samples. K-M survival analysis between high- and low-expression groups of CCNB1 **(G)**, CDCA8 **(H)**, and G6PD **(I)**.*P < 0.05;****P < 0.0001. DEGs, differentially expressed genes.

Finally, we analyzed the three DEGs in the risk signature. The expression of these genes was found to significantly raised in TCGA-HCC samples in comparison with normal tissue (**Figures 9A–C**). These results were confirmed by IHC analysis (**Figures 9D–F**). On the basis of gene expression, patients in the TCGA were classified into one of two groups. Low levels of expression were linked to a better prognosis, thus confirming the results in **Figure 5**, and all three genes represented prognostic risk factors for HCC in the risk signature (**Figures 9G–I**). Moreover, the DEGs expression was revealed to be positively associated with immune cell infiltration using TIMER (**Figures S6A–C**). Boxplots were used to compare the immune cell subset distribution with CNVs, and it can be seen that the greatest differences in arm-level gain in relation to immune infiltration were with CCNB1 and G6PD, whereas the level of immune infiltration with arm-level deletion was most significantly different in CDCA8 (**Figures S6D–F**).

## Discussion

Recent research has shown that the homeostasis of copper is rigorously regulated and that any imbalance reduces the organism’s fitness (45). Disruptions to copper homeostasis have also been linked with tumor growth and irreversible damage (46). Thus, the role of copper *in vivo* has attracted much attention, specifically in the field of tumor therapy. Cuproptosis is a form of programmed cell death; the regulation of cuproptosis is complex, involving numerous regulatory factors. The role of cuproptosis in tumor development and its relationship to immunity has not been fully evaluated. Here, we systematically characterized immune cell infiltration mediated by cuproptosis, as well as the corresponding cuproptosis regulatory subtypes. In addition, a cuproptosis-related risk signature was developed to correlate individual cuproptosis subtypes with the patient’s sensitivity to immunotherapy, suggesting the potential usefulness of the signature for personalized therapy.

The study initially evaluated the expression levels, somatic mutations, and CNVs of 10 cuproptosis regulators, observing that all of the regulatory genes were shown to be expressed differently in tumor and control tissues. The highest mutation frequency was seen in CDKN2A. In addition, the copy numbers of most of the cuproptosis regulators were altered. This suggests that dysfunctional expression of these regulator genes may play an essential role in HCC. Unsupervised consensus cluster analysis of the regulators identified two distinct subtypes in HCC, termed cuproptosis clusters A and B. Surprisingly, GSEA enrichment analysis found that cluster A was mainly related to metabolic processes, while cluster B was enriched in various oncogenic and immune- and stromal-related signaling pathways. Stromal cells are documented to play immunomodulatory roles in tumors (47, 48), are able to prevent the entry of immune cells into the tumor parenchyma (49) and, even if T cells do enter the tumor microenvironment, stromal cells can surround them and prevent an effective immune response (50). Further analysis showed that cuproptosis cluster B was correlated with significant infiltration of immune cells, including regulatory cells such as regulatory T cells and CD 56bright NK cells. These findings suggest that cuproptosis cluster B contains a large number of immune cells, but activity may be suppressed by stromal and immune regulatory cells. K-M survival analysis showed that cluster B was linked with a poorer prognosis, possibly due to tumor escape resulting from immunosuppression.

To quantify the cuproptosis regulatory subtypes in individual tumors, a risk signature based on three DEGs was developed as a scoring system for individual HCC patients in the TCGA cohort. Both K-M survival and ROC curve evaluations verified the signature’s accuracy and reliability for predicting patient prognosis. In addition, we independently verified the signature using gene expression and clinical data from the ICGC database. The signature served as an independent prognostic variable in both the TCGA and ICGC cohorts, as demonstrated by multivariate Cox regression. A nomogram using a combination of the risk signature and clinical features was found to be more effective than other clinical features, greatly improving the clinical utility of the signature.

In our study, three DEGs were identified as cuproptosis-related signature genes. Glucose-6-phosphate dehydrogenase (G6PD) is a rate-limiting enzyme in the pentose phosphate pathway and is involved in energy generation through the maintenance of reduced NADPH co-enzyme levels (51). Because of its key metabolic role, G6PD is also involved in tumor pathogenesis where it has been reported to modulate proliferation (52, 53), metastasis (54), chemoresistance (55, 56), immune activation (57, 58), and tumor ferroptosis (59). Cell division cycle-associated 8 (CDCA8) forms part of the chromosomal passenger complex (CPC) and is necessary for the stabilization of the mitotic spindle. Disruption of cell cycle regulation is a hallmark of tumor development (60). CDCA8 levels are elevated in a variety of tumor types where it has been implicated in the growth and tumor progression (61–64). Cyclin B1 (CCNB1) belongs to the cyclin family and is the regulatory component of cyclin-dependent kinase 1 (65, 66). It has important functions in cell cycle regulation and dysfunctional expression promotes the development of various cancer types, including colon (67–69), cervical cancer (70), and kidney cancer (71). CCNB1 overexpression leads to unplanned entry into the cell cycle, uncontrolled cell proliferation, and tumorigenesis (69, 72–74). Studies have also shown that copper can affect the expression of G6PD and CCNB1, confirming a possible link between DEGs and cuproptosis (75, 76).

Although immunotherapy is effective for HCC (77), its efficacy has not proved consistent due to an incomplete understanding of the immune microenvironment and its variations in individual patients. It is thus necessary to determine which patients are most likely to react to immunotherapy. Here, it was found that the two risk-score subgroups had distinct immune infiltration characteristics with greater infiltration and matrix activity seen in patients with higher risk score. These patients also had lower TIDE scores, suggesting that they were more likely to be sensitive to immunotherapy and less likely to show immune escape (78). Another important result was that there was higher expression of immune checkpoints in this group. And these genes may be targeted by immunotherapy to determine the clinical response of patients (79). The accuracy of the cuproptosis-related risk signature for predicting the response to immunotherapy was confirmed by analysis in the IMvigor210 cohort. In addition, we found differences in TMB values between the groups and demonstrated an association between the risk and TMB scores. It has been reported that higher non-synonymous mutational burdens in tumors result in the formation of greater numbers of neoantigens, leading to greater immunogenicity and enhancing the immunotherapy response (80). This was an additional confirmation of the likelihood that high-risk patients will respond to immunotherapy. These findings indicate that the risk score is both reliable and effective for evaluating cuproptosis-modulating subtypes in individual patients and can also be applied effectively to determining the degree of immune cell infiltration and providing guidance for immunotherapy.

## Conclusion

In conclusion, our study identified two patterns of cuproptosis regulation based on the expression of 10 cuproptosis regulators that are important contributors to the heterogeneity of immune cell infiltration. The cuproptosis-related risk signature, which is closely associated with the prognosis, immune cell infiltration characteristics, and sensitivity to immunotherapy in HCC patients, can quantify the risk of individual patients, providing new directions for individualized anti-tumor immunotherapy for HCC patients.

## Data availability statement

The original contributions presented in the study are included in the article/**Supplementary Material**. Further inquiries can be directed to the corresponding authors.

## Ethics statement

The affiliated hospital of Qingdao University approved the collection of clinical data for research purposes. (Approval No. QYFYWZLL26947). The patients/participants provided their written informed consent to participate in this study.

## Author contributions

GW, RX, and SZ analyzed the data. GW, RX, LS, and JG wrote the manuscript. WL, YZ, and XB reviewed and comprehensively revised the manuscript. WQ and SW contributed to the design of the study. All authors contributed to the article and approved the submitted version.

## Funding

This study was supported by four grants: 1. Beijing Xisike Clinical Oncology Research Foundation (Y-XD2019-11) 2. Qingdao traditional Chinese medicine science and technology project (2021-zyym29) 3. Shandong medical and health science and technology development plan project (202103030554) 4. State Key Laboratory of Ultrasound in Medicine and Engineering (2021KFKT029).

## Acknowledgments

The authors gratefully acknowledge the data provided by patients and researchers participating in TCGA and ICGC.

## Conflict of interest

The authors declare that the research was conducted in the absence of any commercial or financial relationships that could be construed as a potential conflict of interest.

## Publisher’s note

All claims expressed in this article are solely those of the authors and do not necessarily represent those of their affiliated organizations, or those of the publisher, the editors and the reviewers. Any product that may be evaluated in this article, or claim that may be made by its manufacturer, is not guaranteed or endorsed by the publisher.

## Supplementary material

Supplementary Figure 1 Curves of K-M for OS in the groups with high and low expression of 10 cuproptosis regulators. **(A)** FDX1; **(B)** LITP1; **(C)** LIAS; **(D)** DLD; **(E)** DLAT; **(F)** PDHA1; **(G)** PDHB; **(H)** MTF1; **(I)** GLS; **(J)** CDKN2A. K-M Kaplan-Meier, OS overall survival

Supplementary Figure 2 Cuproptosis clustering. **(A–G)** Consensus matrix based on cuproptosis regulator expression in the HCC cohort at k = 2 - 9. **(H)** Consensus CDF plots for cuproptosis clustering. **(I)** The area under the CDF curve relative changes for cuproptosis clustering

Supplementary Figure 3 Gene clustering **(A–G)** Consensus matrix based on cuproptosis-related gene expression in the HCC cohort at k= 2-9. **(H)** Consensus CDF plots for gene clustering. **(I)** The area under the CDF curve relative changes for gene clustering. HCC hepatocellular carcinoma, CDF cumulative distribution function

Supplementary Figure 4 Comparison of risk scores within different grades or between different classifications of clinicopathological features. **(A)** Stage; **(B)**T; **(C)** Grade; **(D)** AFP expression; **(E)** Sex; **(F)** Age.

Supplementary Figure 5 Comparison of drug sensitivity between groups with high and low risk. **(A)** Gefitinib; **(B)** Sorafenib; **(C)** Nilotinib; **(D)** Dasatinib; **(E)** Erlotinib; **(F)** Metformin; **(G)** Bleomycin; **(H)** Doxorubicin; **(I)** Gemcitabine; **(J)** Tipifarnib; **(K)** Imatinib; **(L)** Mitomycin.C.

Supplementary Figure 6 The relationship between the three DEGs in the signature and immunity and copy number. **(A–C)** Correlation between expression of gene and infiltration of immune cell. **(D–F)** Association of gene copy number alterations with infiltration of immune cells. *P < 0.05; **P < 0.01; ***P < 0.001. DEGs differentially expressed genes
