# Supplementary figures and images for: Cuproptosis regulator-mediated patterns associated with immune inﬁltration features and construction of cuproptosis-related signatures to guide immunotherapy
Source: Front Immunol. 2022 Sep 29;13:945516. doi: 10.3389/fimmu.2022.945516 (PMC9559227; doi:10.3389/fimmu.2022.945516)

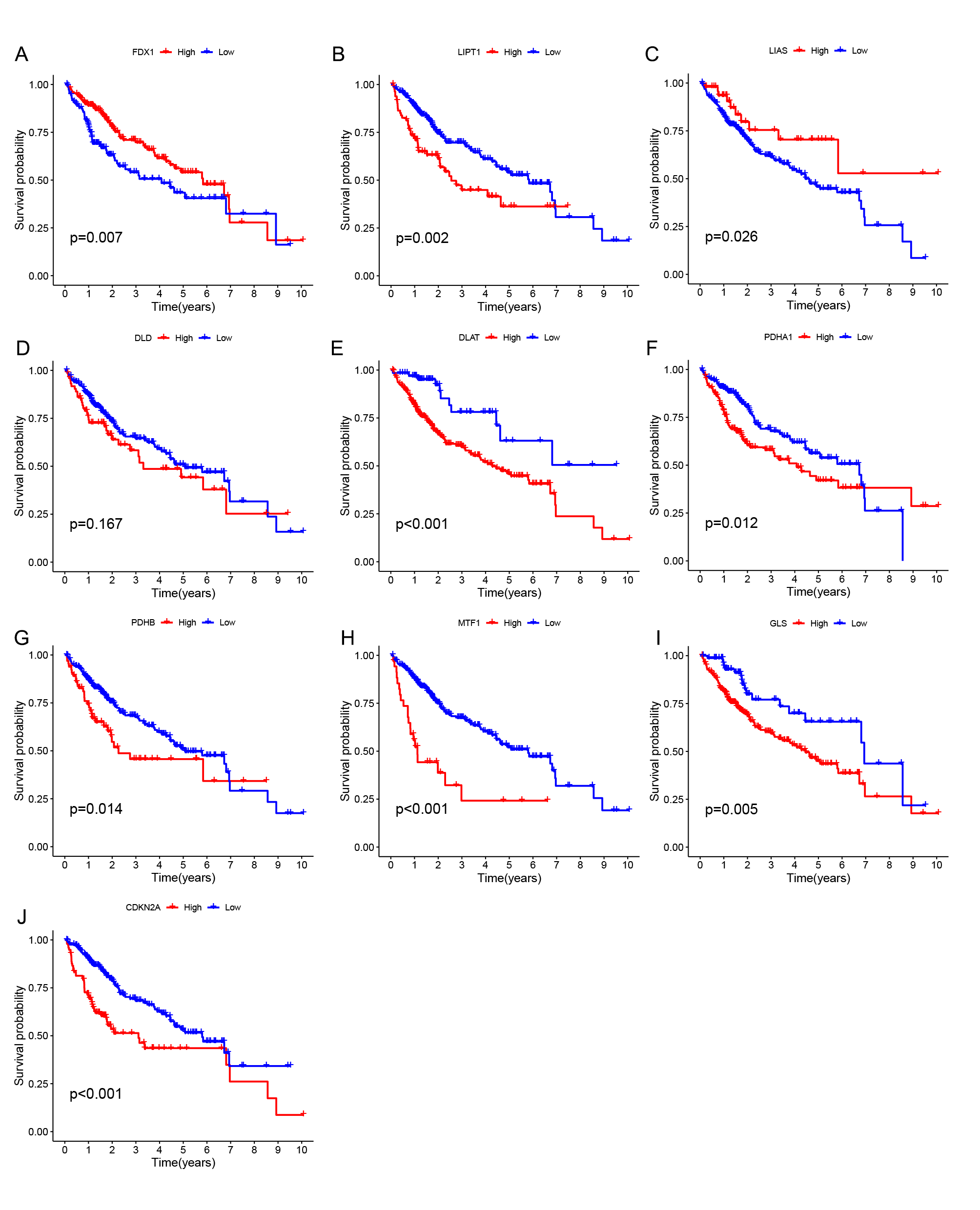

Supplement: Supplementary Figure 1 — Curves of K-M for OS in the groups with high and low expression of 10 cuproptosis regulators. (A) FDX1; (B) LITP1; (C) LIAS; (D) DLD; (E) DLAT; (F) PDHA1; (G) PDHB; (H) MTF1; (I) GLS; (J) CDKN2A. K-M Kaplan-Meier, OS overall survival [file Image_1.tif]

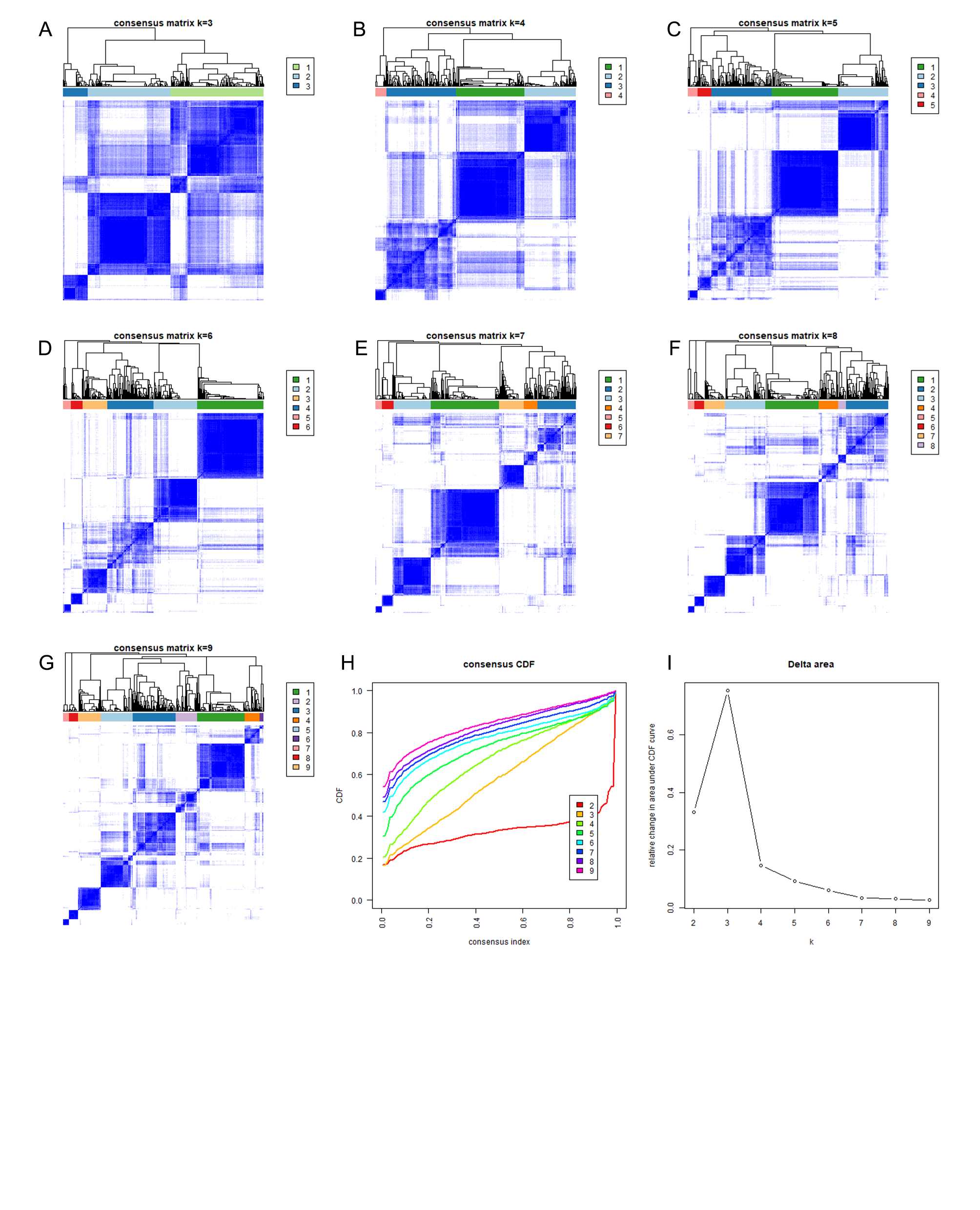

Supplement: Supplementary Figure 2 — Cuproptosis clustering. (A–G) Consensus matrix based on cuproptosis regulator expression in the HCC cohort at k = 2 - 9. (H) Consensus CDF plots for cuproptosis clustering. (I) The area under the CDF curve relative changes for cuproptosis clustering [file Image_2.tif]

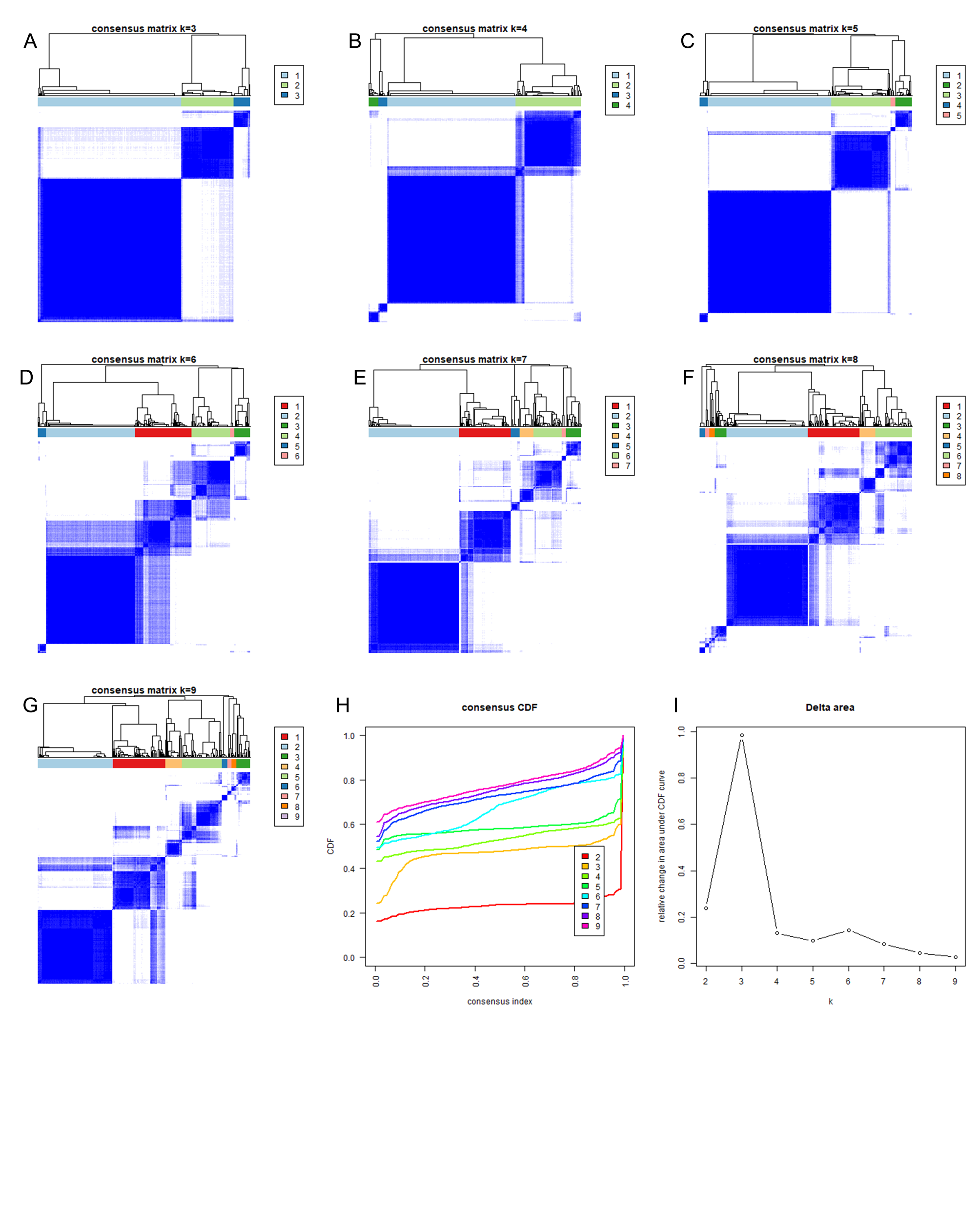

Supplement: Supplementary Figure 3 — Gene clustering (A–G) Consensus matrix based on cuproptosis-related gene expression in the HCC cohort at k= 2-9. (H) Consensus CDF plots for gene clustering. (I) The area under the CDF curve relative changes for gene clustering. HCC hepatocellular carcinoma, CDF cumulative distribution function [file Image_3.tif]

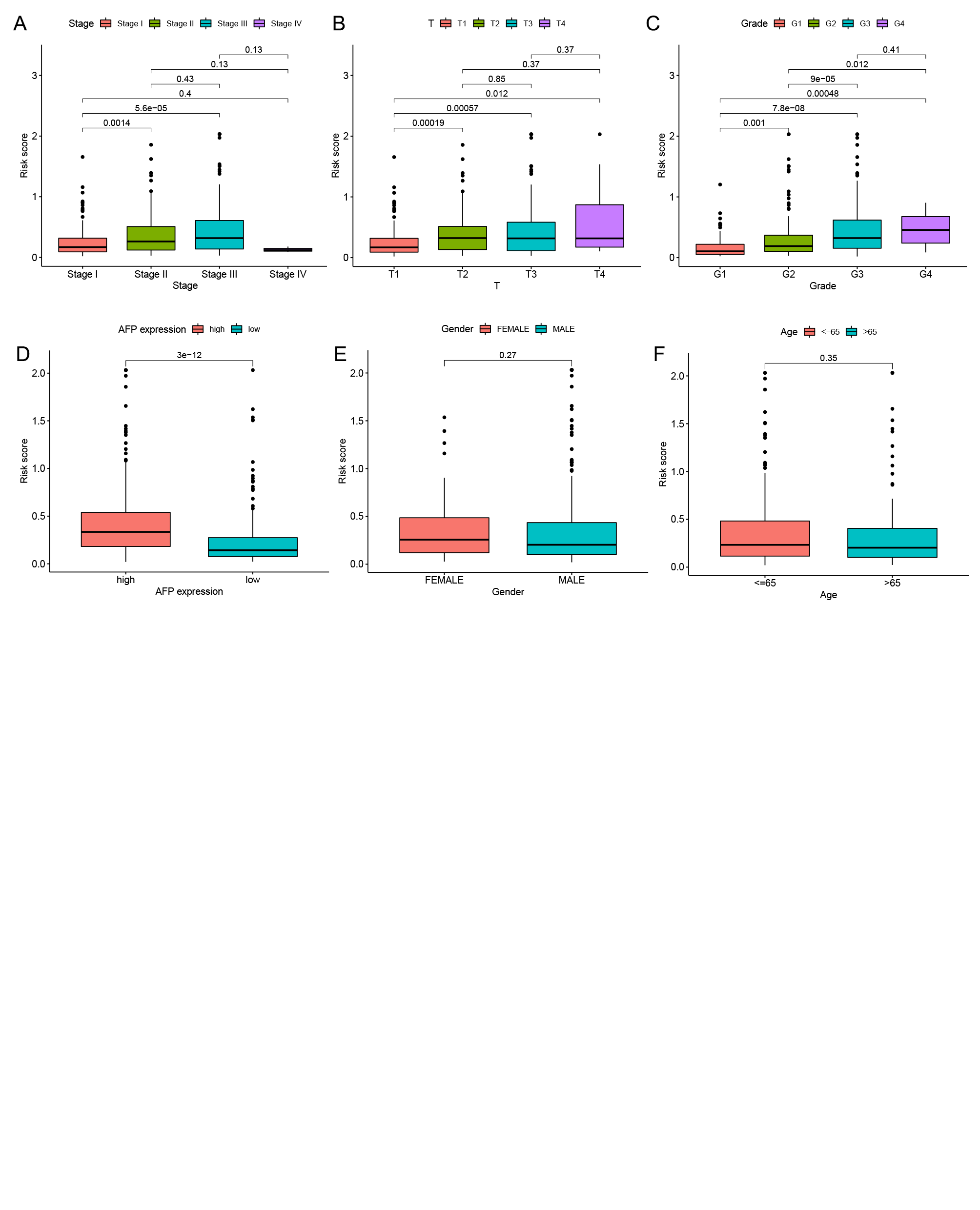

Supplement: Supplementary Figure 4 — Comparison of risk scores within different grades or between different classifications of clinicopathological features. (A) Stage; (B)T; (C) Grade; (D) AFP expression; (E) Sex; (F) Age. [file Image_4.tif]

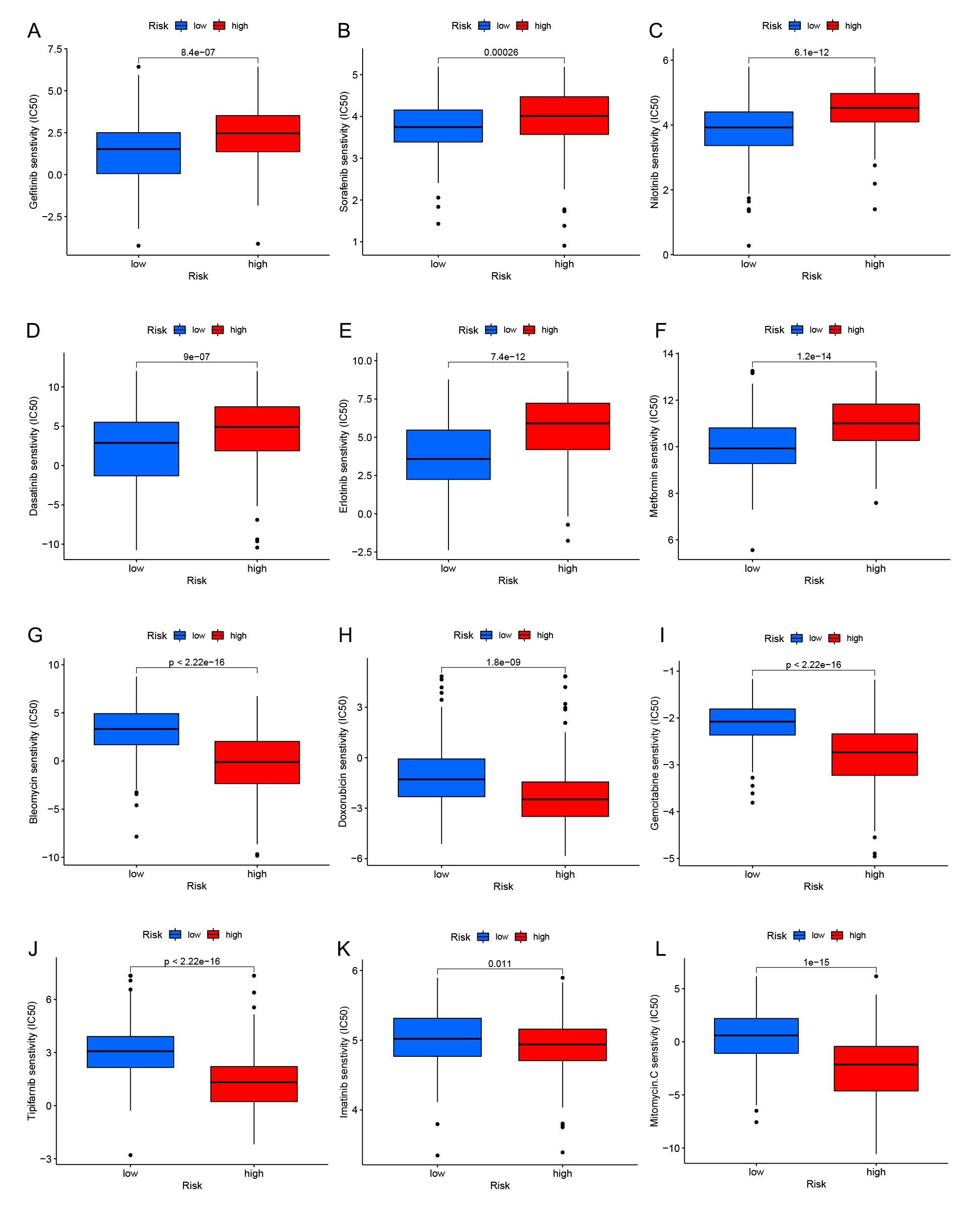

Supplement: Supplementary Figure 5 — Comparison of drug sensitivity between groups with high and low risk. (A) Gefitinib; (B) Sorafenib; (C) Nilotinib; (D) Dasatinib; (E) Erlotinib; (F) Metformin; (G) Bleomycin; (H) Doxorubicin; (I) Gemcitabine; (J) Tipifarnib; (K) Imatinib; (L) Mitomycin.C. [file Image_5.tif]

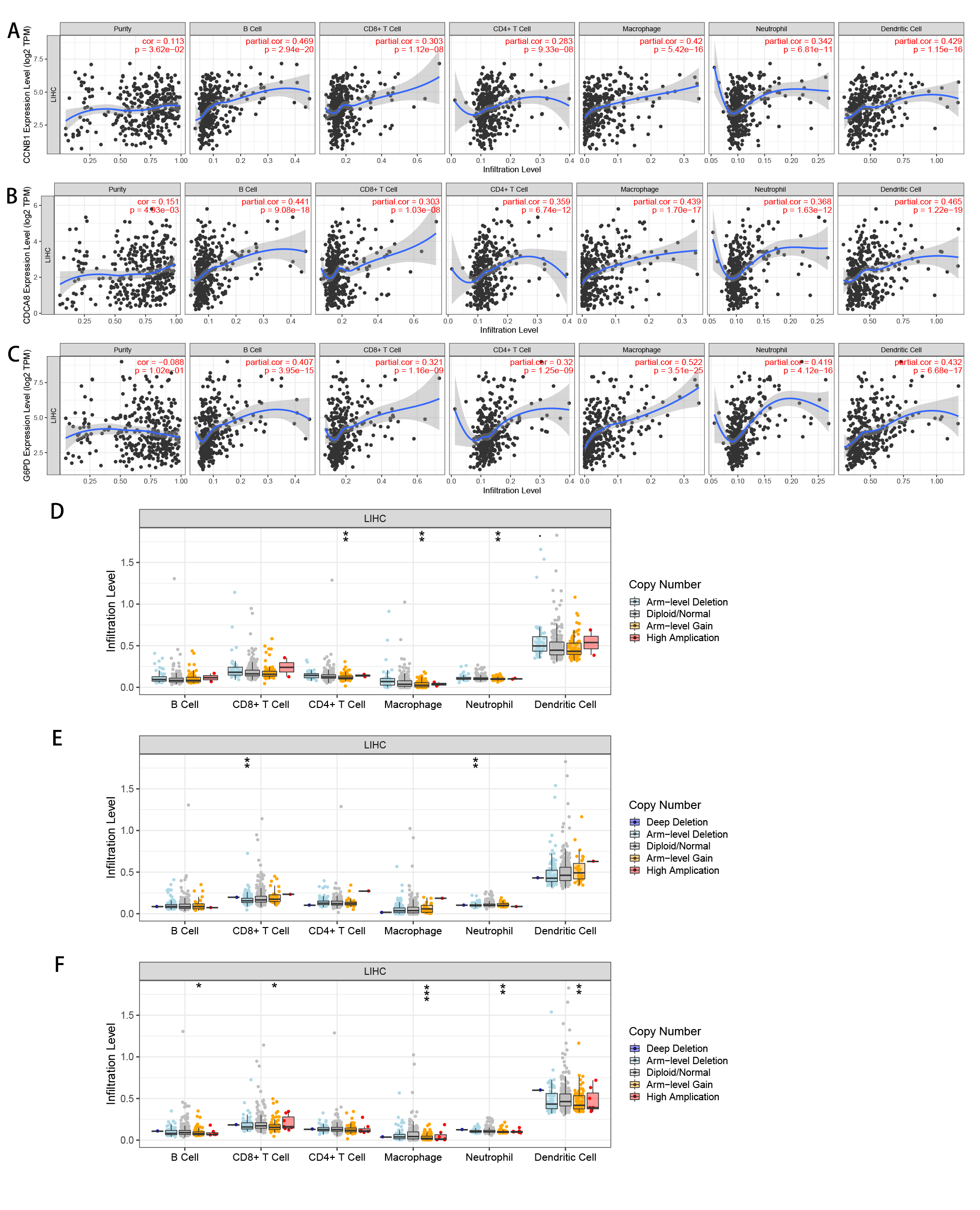

Supplement: Supplementary Figure 6 — The relationship between the three DEGs in the signature and immunity and copy number. (A–C) Correlation between expression of gene and infiltration of immune cell. (D–F) Association of gene copy number alterations with infiltration of immune cells. *P < 0.05; **P < 0.01; ***P < 0.001. DEGs differentially expressed genes [file Image_6.tif]
