# Supplementary material for: Cuproptosis regulator-mediated patterns associated with immune inﬁltration features and construction of cuproptosis-related signatures to guide immunotherapy
Source: Front Immunol. 2022 Sep 29;13:945516. doi: 10.3389/fimmu.2022.945516 (PMC9559227; doi:10.3389/fimmu.2022.945516)
Supplement: Supplementary file 13 [file Table_7.docx]

https://www.jianguoyun.com/p/DbwbKKwQ5ujMChjq0b8EIAA
